# Subchronic and Genetic Safety Assessment of a New Medicinal* Dendrobium* Species:* Dendrobium* Taiseed Tosnobile in Rats

**DOI:** 10.1155/2018/8950534

**Published:** 2018-02-06

**Authors:** Li-Chan Yang, Jiunn-Wang Liao, Chi-Luan Wen, Wen-Chuan Lin

**Affiliations:** ^1^Department of Pharmacy, School of Pharmacy, China Medical University, Taichung, Taiwan; ^2^Graduate Institute of Veterinary Pathobiology, National Chung Hsing University, Taichung, Taiwan; ^3^Taiwan Seed Improvement and Propagation Station, Council of Agriculture, Taichung, Taiwan

## Abstract

*Dendrobium* Taiseed Tosnobile is a new species of herba dendrobii (Shi-Hu) that was developed by crossbreeding* D. tosaense* and* D. nobile*. Its pharmacological activity and active component have been reported, but its subchronic toxicity and genetic safety have not yet been investigated. This study assessed the 90-day oral toxicity and genetic safety of the aqueous extracts of* D.* Taiseed Tosnobile (DTTE) in male and female Sprague-Dawley (SD) rats. Eighty rats were divided into four groups, each consisting of ten male and ten female rats. DTTE was given orally to rats at 800, 1600, or 2400 mg/kg for 90 consecutive days, and distilled water was used for the control group. Genotoxicity studies were performed using a bacterial reverse mutation assay and in vivo mammalian cell micronucleus test in ICR mice and analyzed using flow cytometry. Throughout the study period, no abnormal changes were observed in clinical signs and body weight or on ophthalmological examinations. Additionally, no significant differences were found in urinalysis, hematology, and serum biochemistry parameters between the treatment and control groups. Necropsy and histopathological examination indicated no treatment-related changes. Based on results, the no-observed-adverse-effect level of DTTE is greater than 2400 mg/kg in SD rats.

## 1. Introduction

The raw pharmacognosy of herba dendrobii (Shi-Hu), a medicinal orchid (family: Orchidaceae), is well known; it has been widely used for health improvement as an herbal tea, and it is also included in traditional Chinese and other Asian folk medicines for its antipyretic, ophthalmic, and tonic benefits [1, 2]. Unlike other medicinal material herbs that usually comprise only one species, herba dendrobii is prepared from several* Dendrobium* species—namely,* D. tosaense*,* D. huoshanense*,* D. nobile, *and* D. candidum *[1, 3, 4]. Herba dendrobii is highly valuable, but its growth rate is slow and its distribution in the wild is sparse; therefore, it is a protected herb before. Recently, researchers have attempted to improve the large-scale cultivation of herba dendrobii. One solution was to develop a new giant-type species with a higher growth rate.* D.* Taiseed Tosnobile is a new species developed by crossbreeding* D. tosaense* and* D. nobile* as the seed parent and the pollen parent, respectively, by the Taiwan Seed Improvement and Propagation Station [4]. It exhibits the characteristics of high yield and easy cultivation and has no special pest control requirements.

Numerous studies have reported the structural characteristics and some pharmacological properties of the polysaccharides of other* Dendrobium* species, which include antitumor, antihyperglycemic, antioxidant, hepatoprotective, epithelial repairing, and immunomodulatory activities [4–9]. Among the pharmacological activities of the polysaccharides of dendrobium, immunomodulatory activity is the most common activity. As a new species of herba dendrobii, demonstrating the functional components, pharmacologic properties, and safety is necessary. Yang et al. (2017) reported that polysaccharides from aqueous extracts of *D*. Taiseed Tosnobile have immunostimulating action* in vivo*. Additionally, no safety studies on *D*. Taiseed Tosnobile have been conducted. In tradition, the way that people use herba dendrobii is by extracting the stem with boiling water. Therefore, this study demonstrated the safety of aqueous extracts of D. Taiseed Tosnobile, but not other components from *D*. Taiseed Tosnobile to meet the general mode of use. Therefore, to better understand the availability of this new* Dendrobium* species, we performed a 90-day subchronic toxicity study of the oral administration of the aqueous extract of *D*. Taiseed Tosnobile (DTTE) and conducted a genetic safety assessment using the bacterial reverse mutation assay and micronucleus test in vivo.

## 2. Materials and Methods

### 2.1. Material Preparation

The plant material of *D*. Taiseed Tosnobile was provided by the Taiwan Seed Improvement and Propagation Station (Taichung, Taiwan). The dry stems were extracted by 95°C distilled water over 1 h, and then the aqueous extracts were evaporated under reduced pressure to decrease the volume. DTTE was stored at −20°C until used and the yield rate was 20.3% in dry stems. The yields of crude polysaccharides were 8.3% for dry stems. The moisture of DTTE was 26.3%. The composition analysis of DTTE is shown in Table 1. DTTE includes calories, crude protein, crude fat, saturated fatty acids, trans fatty acids, and carbohydrate according to the Association of Official Agricultural Chemists (AOAC) official procedures [10].

### 2.2. 90-Day Oral Exposure Study

#### 2.2.1. Animals and Treatment

This 90-day subchronic toxicity study was designed to evaluate oral exposure of DTTE according to the Organization for Economic Cooperation and Development (OECD) guideline 408. Animal experimental protocols complied with the institutional guidelines of China Medical University for the use of laboratory animals. The guidelines corresponded to the National Institutes of Health guide for the care and use of laboratory animals (NIH Publications number 8023, revised 1978). Six-week-old Sprague-Dawley (SD) rats were purchased from BioLASCO Taiwan Co., Ltd. (Yilan, Taiwan), and were acclimated for 2 weeks before beginning the experiment. The initial body weight of the rats was 247 ± 7 g and 187 ± 7 g for the males and females, respectively. The rats were housed in an air-conditioned room (21–24°C) with humane care and 12 h of light daily (8:00 a.m. to 8:00 p.m.); they were also allowed free access to food pellets and water throughout the study period. The rats were randomly divided into the control and treatment groups of ten rats per sex per group. DTTE was given to the experimental rats daily by gavage at different doses—800, 1600, or 2400 mg/kg—and distilled water was given to the control group. To prevent adverse effects from gavage, the volume of DTTE in different dosage and water was calculated to be 10 mL/kg. During the experimental period, all animals were observed twice daily for mortality, morbidity, and clinical signs of toxicity. Body weight was measured pretest, weekly, and on the day of sacrifice after overnight fasting. Moreover, food consumption was determined weekly. At the end of the experiment, all surviving animals were anesthetized with isoflurane (Piramal Critical Care, PA, USA) and euthanized after blood collection.

#### 2.2.2. Urinalysis

During the last week of the study, each surviving rat was placed in a metabolic cage for 16 h to collect urine. The appearance, color, and volume of the urine samples were determined, and they were also analyzed for pH, specific gravity, leukocytes, occult blood, nitrite, protein, glucose, ketones, urobilinogen, and bilirubin with an autoanalyzer (Urisys 1100, Roche, Switzerland). Sediments of each urine sample were microscopically examined for leukocytes, red blood cells (RBCs), epithelial cells, microbes, crystals, and casts.

#### 2.2.3. Hematology and Serum Biochemistry

After overnight fasting, all surviving rats received anesthesia and were subject to bloodletting for euthanization. The blood was examined for blood sugar immediately using a commercial glucose meter with strips (Accu-Chek, Roche, Germany). The blood was collected in a spray-dried K_3_EDTA blood collection tube (Greiner Bio-One, Austria) and mixed well. A complete blood count (CBC) counter was applied to the following detection: erythrocytes, hemoglobin, hematocrit, mean corpuscular volume (MCV), mean corpuscular hemoglobin (MCH), mean corpuscular hemoglobin concentration (MCHC), platelets, and leucocytes. Then, a blood smear was prepared and stained using the commercial Diff-Quick Stain kit (Sysmex Corporation, Japan) for the microscopic detection of lymphocytes and neutrophils. If effects on the hematopoietic system were noted, reticulocyte count and terminal bone marrow cytology were examined microscopically. Further, 1.8 mL of blood sample was placed in a coagulation tube containing 3.2% sodium citrate (Becton Dickinson, USA) and then centrifuged at 1500*g* for 15 min to separate the serum, which was analyzed for the prothrombin time (PT) and the activated partial thromboplastin time (APTT). Other blood samples were collected in blood collection tubes containing *Z* serum clot activator (Greiner Bio-One), after which the serum was separated. The following clinical chemistry parameters were analyzed using an automatic biochemical analyzer (Cobas Mira Plus, Roche, Germany): total protein (TP), albumin (ALB), aspartate aminotransferase (AST), alanine aminotransferase (ALT), alkaline phosphatase (ALP), total bilirubin (TBIL), gamma-glutamyl transferase (GGT), creatinine (CREA), blood urea nitrogen (BUN), and electrolytes, including sodium, potassium, chloride, calcium, and phosphate.

#### 2.2.4. Pathology

At the end of the treatment, all surviving animals were fasted overnight and sacrificed the following day by exsanguination and euthanization. Before sacrifice, the rats were weighed and external and internal gross pathological examinations were performed. The weights of major organs such as the brain, heart, kidney, liver, spleen, adrenal gland, testes, and ovaries were recorded after the removal of peripheral fat tissue. In addition, relative organ weights were calculated against the fasting body weight. The peripheral oral cavity, cranial cavity, and all tissues and organs in the thoracic and abdominal cavities were examined visually for any abnormality. Histopathological examinations were performed on the brain, heart, kidney, liver, spleen, adrenals, testes, ovaries, aorta, bone marrow, duodenum, jejunum, ileum, caecum, colon, rectum, eyes, esophagus, mammary gland, Harderian gland, trachea, lung, lymph node, pancreas, sciatic nerve, pituitary, prostate gland, salivary gland, skin, spinal cord, stomach, thigh muscle, thymus, thyroid/parathyroid gland, urinary bladder, and uterus. Except for the testes and eyes, all of the aforementioned collected tissues were fixed in 10% neutral buffered formalin (Tonyar Biotech, Taiwan). The lungs were inflated with a fixative prior to immersion in the formalin. The eyes and testes were fixed in modified Davidson's Fluid for 48 hours and then briefly washed in tap water before being transferred to the 10% neutral buffered formalin for storage prior to trimming and processing [11]. Preserved organs and tissues were dehydrated, clarified, infiltrated with paraffin, and embedded after trimming to form paraffin tissue blocks, which were sliced into 5 *μ*m thick sections using a microtome (Leica RM 2145, Germany); these sections were stained with hematoxylin and eosin (HE). Histopathological analysis was conducted using an optical microscope (Optiphot-2; Nikon, Japan). All tissues from animals in the control and high-dose groups were examined, with gross lesions examined microscopically. If treatment-related effects were noted in certain tissues, we evaluated the tissue samples of the rats at the next lower dose. Successive examination of the next lower doses continued until no effects were noted. We also performed microscopic examinations of all tissues from all the animals (both those that died prematurely and those sacrificed at the end of the study) to assess any potential toxic effects.

### 2.3. Genotoxicity Studies

#### 2.3.1. Bacterial Reverse Mutation Assay (Ames Test)

Bacterial mutation assay was performed according to* Health Effects Test Guidelines*,* Bacterial Reverse Mutation Test* [12], and* OECD Guidelines for the Testing of Chemicals, *Section 4: Health Effects,* number 473: Bacterial Reverse Mutation Test* [13]. Strains TA98 and TA100 of the histidine-requiring* Salmonella typhimurium* were purchased from the Bioresources Collection and Research Center (FIRDI, Hsinchu, Taiwan) and strains TA102, TA1535, and TA1537 were purchased from Discovery Partners International (DPI, USA). The genotypes of the bacterial strains were confirmed by histidine mutation, rfa mutation, uvrB repair, and ampicillin resistance before the assay; also prior to the assay, a dose range-finding test was performed with DTTE (1.25, 2.5, and 5 mg/plate) in the tester strains. A plate incorporation assay was employed and performed to detect a reverse mutation in bacterial strains. Briefly, 0.1 mL of aqueous solution of DTTE (0.313, 0.625, 1.25, 2.5, and 5 mg/plate) was mixed with 0.1 mL of overnight culture of the* S. typhimurium* strains (2 × 10^9^ cells/mL) in 0.5 mL of 0.2 M phosphate buffer either without or with the S9 metabolic activation mix (0.5 mL). The composition of the S9 mixture was 5%* v/v* Aroclor-1254 that had been induced in rat livers (Lot#2901, MoltoxTM, USA) and 0.15 M KCl (Sigma-Aldrich, USA). The S9 mixture was subsequently mixed with 2 mL of molten top agar solution with 0.5 mM histidine/biotin. Next, the cultures were incubated at 50°C ± 1°C before being transferred to minimal-glucose agar plates. The solidified agar plates were inverted and incubated at 37°C ± 1°C for 48–72 h; then, the colonies were counted. Double distilled water served as the negative control. Positive reagents without S-9 mix reactions were 2.5 *μ*g/plate 4-nitroquinoline-N-oxide for TA98, 5 *μ*g/plate sodium azide for TA100, TA1535, and 0.5 *μ*g/plate mitomycin C for TA102, and 50 *μ*g/plate 9-aminoacridine for TA1537; positive reagent with S-9 mix reactions was 5 *μ*g/plate 2-aminoanthracene for all* Salmonella* strains.

#### 2.3.2. In Vivo Mammalian Cell Micronucleus Test

The micronucleus test was performed as described by Bemis et al. (2008) using a commercial MicroFlowPLUS Kit (Becton Dickinson). Twelve-week-old male ICR mice (body weight, 25–30 g) were purchased from BioLASCO Taiwan Co., Ltd. All positive control group mice were given 100 mg/kg cyclophosphamide via intraperitoneal injection (10 mL/kg); then, the test sample groups received DTTE orally at 800, 1600, or 2400 mg/kg (dosed at 10 mL/kg). The negative control group received sterile water at 10 mL/kg. All doses were administered using a stainless steel feeding needle. The mice were monitored daily for any posttreatment clinical symptoms, and their body weight before and after treatment was noted. At 24 and 48 h posttreatment, 60 *μ*L of blood was collected in a K_3_EDTA tube (BD) by tail vein bleeding. Blood samples were then fixed with ultra-cold methanol and were kept for at least 48 h at −80°C for use in flow cytometric analysis. Fixed blood cells were washed and centrifuged, and the washed cells were stained with a labeled solution containing RNase, anti-CD71-FITC (BioLegend), and anti-CD61 PE for 30 min at 4°C. Next, the samples were moved to room temperature and incubated for 30 min at room temperature to ensure the complete degradation of cellular RNA. Then, all samples were kept on ice until analysis. Samples were suspended by gently tapping the tube, adding 1 mL of DNA staining solution (propidium iodide, PI), and placing the tube on the cytometer. The CD71^−^ mouse blood sample, PI^−^ blood sample, and CD61^−^ blood sample were used for compensation before flow cytometry analysis (Becton Dickinson FASCScan, Mountain View, USA). Reticulocytes (RETs) are CD71^+^ cells, and at least 20,000 CD71^+^ RETs were collected for each sample; % RET = CD71^+^ cells/total cells. MN-RETs are CD71^−^ and PI-positive cells and were calculated as follows: (CD71^+^ + PI^+^)/CD71^+^. Normal RBC cells were depicted as CD71^−^/PI^−^.

### 2.4. Statistical Analysis

All data were expressed as mean ± standard deviation. The analyses of body weight, feed consumption, organ weight, hematology, and serum biochemistry were performed with one-way analysis of variance using PASW Statistics software (IBM, USA). Duncan's test was used to determine the statistical significance (*P* < 0.05) between the control and treatment groups, and male and female rats were evaluated separately.

## 3. Results

### 3.1. 90-Day Oral Exposure Study

#### 3.1.1. Clinical Observation, Feed Consumption, and Body Weight

**Figure 1:**
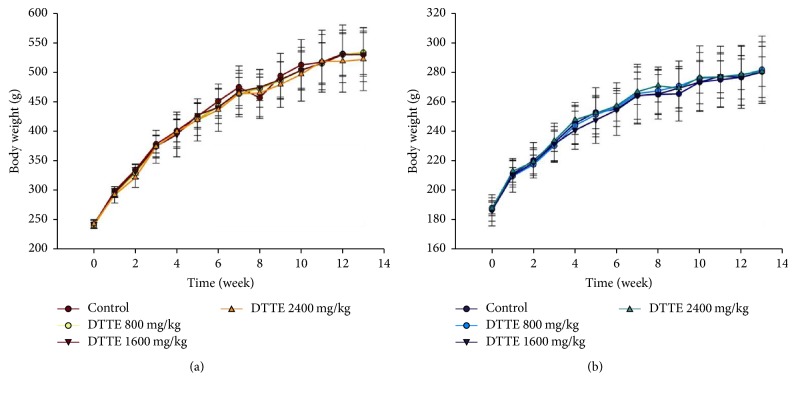
The body weight change of SD rats during the administration of DTTE for 90 days. (a) Male rats and (b) female rats. Data are expressed as the mean ± SD (*n* = 10).

No rats died, and no clinical signs of toxicity or mortality related to DTTE administration at any dose were observed in the rats during this 90-day subchronic study. Food consumption between the DTTE-treated groups and the control group was the same (Figure 1). Compared with the control group, no significant influences were observed in the DTTE-treated male and female groups (Figure 1). Ophthalmoscopic examinations revealed no abnormality in the DTTE-treated or control groups.

#### 3.1.2. Urinalysis

No significant differences in urine sediments or urinalysis response variables were observed between the treatment and control groups of both sexes (data not shown).

#### 3.1.3. Hematology and Serum Biochemistry

The platelet count in male rats who received 2400 mg/kg DTTE was significantly lower than that in the control group (Table 2); however, the platelet count was still within the normal range, which is verified by a previous report.

After 90 days, the lymphocyte count and segmented neutrophil count in female rats given DTTE 800 mg/kg were lower and higher than those in the control group, respectively. However, the lymphocyte count was not significantly different between the control and low-dose groups (5.8 ± 0.5 and 6.1 ± 0.8 × 10^3^/*μ*L, resp.). The segmented neutrophil count in the low-dose DTTE-treated females (0.59 ± 0.11 × 10^3^/*μ*L) did not significantly increase compared to that in the control group (0.74 ± 0.08 × 10^3^/*μ*L). Results showed that the statistical differences observed in one sex were not observed in the other and were found to be non-dose-dependent. No other hematological parameters in either sex were significantly different compared to those in the controls.

Table 3 shows the results of the serum biochemistry analysis. Serum sodium among the high-dose group was significantly higher than that of the control group in male rats, whereas serum chloride among the high-dose group was significantly higher than that of the control group in female rats. Notably, both serum electrolytes were still within the normal range, according to a previous report [14]. Other small yet significant changes following DTTE administration were observed in the serum biochemistry parameters and were considered to be incidental changes (i.e., biological variations) and not treatment-related adverse effects.

#### 3.1.4. Pathology

No treatment-related gross pathological changes were observed in any group. Moreover, no treatment-related changes were observed in tissues of the peripheral cavity, thymus, heart, lung, liver, kidney, gastrointestinal tract, spleen, brain, and the reproductive system in the treatment or control groups (Table 4), and no significant intersex difference was observed for absolute organ weight compared with the control group. Additionally, no significant differences were found in the relative organ weight between either sex or the control group (data not shown).

The histopathological examination was performed on 40 rats (ten rats per sex from the control and high-dose group), and the results are shown in Table 5. No significant treatment-related changes were observed in either the DTTE-treated or control groups among the males and females. In males, slight mononuclear cell infiltration (focal) was found in the heart, with the incidence rate of this phenomenon in the control and high-dose group 2/10 and 1/10, respectively. Slight to moderate focal hydronephrosis and focal mononuclear cell infiltration were found in the kidney of the male control group, and the incidence rate of these phenomena was 2/10 and 1/10, respectively. Notably, only one male rat in the control group present focal slight tubular regeneration in the kidneys. Both control and high-dose groups had focal, slight, mononuclear cell infiltration in the prostate gland, and the incidence rate of this phenomenon was 2/10 for both groups. Only one female rat in the high-dose DTTE-treated group presented with a focal and moderate epidermoid cyst in the stomach. Though some differences were observed, these results were not considered relevant to the test article treatment (Table 5).

### 3.2. Genotoxicity Studies

#### 3.2.1. Bacterial Reverse Mutation Assay

Before the Ames test, all strains were treated with DTTE at 1.25, 2.5, or 5 mg/plate for 18 h to evaluate the toxicity effects (Table 6). The results showed that DTTE had no significant toxic effect on the strains at any of the administered doses. In the Ames test, for which DTTE was directly applied to the strain at 0.3125, 0.625, 1.25, 2.5, or 5 mg/plate for 48 h with and without S9 mix, the number of mutant bacteria was not more than 2-fold higher than in the negative control group in both the presence and absence of S9 mixture (Table 7). Thus, DTTE did not induce mutations in the* Salmonella* reverse mutation assay.

#### 3.2.2. In Vivo Mammalian Cell Micronucleus Test

Cyclophosphamide is a known mutagenic agent that affects RBC metabolism and causes double-strand breaks in DNA. Our results showed that the ratio of micronucleated (MN) RBCs to RETs was significantly increased in the cyclophosphamide-treated group compared with that in the control group after 24 and 48 h of treatment. Furthermore, the ratio of RETs to normochromatic erythrocytes (NCEs) in the cyclophosphamide-treated group was significantly decreased compared with that in the control group after 24 and 48 h of treatment (Table 8). As for DTTE-treated groups, there was no significant difference compared with the control group in the ratio of MN/RETs or RETs/NCEs. The micronucleus test showed that DTTE did not affect the number of peritoneal MN RBCs or the production of RETs in mice.

## 4. Discussion

We evaluated the safety of DTTE in a 90-day subchronic study of oral toxicity and two genotoxicity studies of mammalian and bacterial systems and found that DTTE did not cause any general organ or systemic toxicity in low- or high-dose-treated rats. The results of the* Salmonella* reverse mutation assay and the micronucleus test in mice demonstrated that DTTE is not mutagenic or genotoxic.

During the 90-day subchronic toxicity, there were no mortalities and no changes in the ophthalmological status, clinical status, body weight, food consumption, or food efficiency that were attributable to DTTE administration. Some isolated significant changes were observed in certain hematological and biochemical parameters in the DTTE-treated groups. However, these changes did not occur in both sexes and were not dose-dependent. Although the platelet count in the males of the 2400-mg/kg-DTTE-treated group was significantly different than that in the control group, the value was still within the normal range [14] Nevertheless, the ratio of lymphocytes to segmented neutrophils in 800 mg/kg DTTE-treated group in females significantly differed from the control group, but the lymphocyte and segmented neutrophil counts in the control and low-dose groups were not different. Additionally, biochemistry tests showed that sodium concentration in the high-dose group in males and the chloride concentration in the high-dose group in females were higher than the respective values in the control group, but still within the normal range [14]. No significant changes were found through the urinalysis, and the histopathological examination showed only slight mononuclear cell infiltration in the heart and prostate in the control and treatment groups in males and an epidermoid cyst in the stomach of one female rat in the treatment group. No positive correlations between the degree and incidence of the changes between the treatment and control groups were noted, and the histopathological changes were nonspecific.

Some previous studies have reported the toxicity evaluations of other *D*. species, such as* D. officinale*,* D. candidum and D. moniliforme *[15–17]. Lee et al. [15] investigated the acute toxicity of* D. moniliforme* aqueous extract by a single oral dose in Sprague-Dawley rats and found out that* D. moniliforme* aqueous extract did not induce any toxic effects in rats for both sexes. The genetic toxicity test and 90-day feeding test in rats of* D. candidum *are performed and results indicate that the aqueous extracts were without toxicity, genetic toxicity, and mutagenicity under the test dose [16]. In addition, the genetic test of* D. officinale* shows negative results [17]. Although previous toxicology studies of herba dendrobii show that some *D*. species are nontoxic, we though it is necessary to investigate a safety assessment of a new medicinal Dendrobium species,* D.* Taiseed Tosnobile. In this study, the outcomes of the present subchronic toxicity study showed that oral administration of DTTE at concentrations of up to 2400 mg/kg does not cause adverse effects in male or female rats and that DTTE does not cause any genotoxic effects. Thus,* D. *Taiseed Tosnobile, a new* Dendrobium* species, could be safe for possible use as an herb or herbal tea, provided that the margin of safety between the no-observed-adverse-effect level of 2400 mg/kg and estimated intake resulting from the proposed uses and use levels are adequate.

## Figures and Tables

**Table 1:** Composition analysis of DTTE.

Items	Content (per 100 g)
Calories	425.7 kcal
Crude protein	3.2 g
Crude fat	18.1 g
Saturated fatty acids	2.0 g
Trans fatty acids	Trace
Carbohydrate	62.5 g
Sodium	21 mg
Moisture	26%

**Table 2:** Hematology parameters of SD male and female rats fed with 800, 1600, and 2400 mg/kg DTTE for 90 days.

Parameters	Control	800 mg/kg	1600 mg/kg	2400 mg/kg
Erythrocytes (10^6^/*μ*L)				
M	8.2 ± 0.5	8.3 ± 0.5	8.3 ± 0.3	8.3 ± 0.4
F	7.2 ± 0.1	7.4 ± 0.1	7.4 ± 0.1	7.4 ± 0.1
Hemoglobin (g/dL)				
M	15.2 ± 0.6	15.5 ± 0.5	5.7 ± 0.5	15.2 ± 0.6
F	14.6 ± 0.1	15.1 ± 0.3	15.0 ± 0.2	14.7 ± 0.2
Hematocrit (%)				
M	45.5 ± 2.5	46.5 ± 2.2	46.9 ± 1.4	46.3 ± 2.1
F	42.1 ± 0.6	43.5 ± 0.7	43.3 ± 0.4	43.1 ± 0.6
MCV (*μ*^3^)				
M	55.5 ± 1.1	56.2 ± 1.9	56.0 ± 1.6	56.1 ± 1.5
F	58.4 ± 0.3	58.6 ± 0.5	58.5 ± 0.5	58.7 ± 0.6
MCH (pg)				
M	18.6 ± 0.6	18.7 ± 1.0	18.8 ± 0.8	18.4 ± 0.5
F	20.2 ± 0.2	20.4 ± 0.2	20.3 ± 0.2	20.0 ± 0.3
MCHC (g/dL)				
M	33.5 ± 0.6	33.2 ± 0.8	33.4 ± 0.7	32.9 ± 0.3
F	34.5 ± 0.2	34.7 ± 0.2	34.7 ± 0.2	34.0 ± 0.2
Platelets (10^3^/*μ*L)				
M	1185 ± 108	1107 ± 159	1109 ± 125	996 ± 238^*∗*^
F	1108 ± 49	1074 ± 52	1160 ± 45	1132 ± 42
Leukocytes (10^3^/*μ*L)				
M	10.7 ± 2.6	8.4 ± 2.2	10.4 ± 4.6	9.0 ± 1.5
F	6.3 ± 0.5	6.9 ± 0.9	7.1 ± 0.4	6.2 ± 0.6
Lymphocytes (%)				
M	88.5 ± 2.9	89.2 ± 2.6	83.8 ± 18.3	87.1 ± 4.4
F	91.9 ± 0.9	88.9 ± 0.8^*∗*^	90.6 ± 0.5	90.7 ± 0.6
Seg. Neu (%)				
M	11.5 ± 2.9	10.8 ± 2.6	16.2 ± 18.3	13.0 ± 4.4
F	8.1 ± 0.9	11.2 ± 0.8^*∗*^	9.4 ± 0.5	9.3 ± 0.6
PT (sec)				
M	10.0 ± 0.2	10.4 ± 1.2	10.1 ± 0.4	10.0 ± 0.2
F	10.8 ± 0.5	11.0 ± 0.4	11.0 ± 0.8	11.5 ± 1.4
APTT (sec)				
M	17.4 ± 0.8	18.6 ± 2.6	18.8 ± 1.8	17.7 ± 0.7
F	23.7 ± 2.3	21.0 ± 1.9^*∗*^	22.1 ± 2.6	24.2 ± 2.8

All values are means ± SD. ^*∗*^*P* < 0.05 as compared with the control group; M: male; F: female; and Seg. Neu: segmented neutrophil.

**Table 3:** Clinical biochemistry of SD male and female rats fed with 800, 1600, and 2400 mg/kg DTTE for 90 days.

Parameters	Control	800 mg/kg	1600 mg/kg	2400 mg/kg
ALP (U/L)				
M	50.7 ± 7.7	50.7 ± 11.9	53.1 ± 7.5	54.1 ± 9.1
F	29.2 ± 5.7	25.7 ± 3.6	28.3 ± 5.4	31.0 ± 10.0
AST (U/L)				
M	59.1 ± 7.8	58.5 ± 6.9	54.5 ± 8.3	66.6 ± 7.3
F	62.0 ± 9.9	84.7 ± 17.0	81.2 ± 13.3	63.1 ± 9.7
ALT (U/L)				
M	37.3 ± 5.8	38.7 ± 5.1	35.3 ± 3.3	43.2 ± 16.2
F	34.0 ± 8.2	28.2 ± 3.3	32.2 ± 4.3	40.3 ± 5.6
*γ*-GT (IU/L)				
M	2.7 ± 1.6	2.9 ± 2.4	2.6 ± 2.1	1.6 ± 1.4
F	2.3 ± 1.3	2.9 ± 2.6	2.1 ± 1.3	2.1 ± 1.0
T-protein (g/dL)				
M	6.1 ± 0.2	6.1 ± 0.2	6.1 ± 0.2	6.1 ± 0.3
F	6.4 ± 0.2	6.5 ± 0.2	6.4 ± 0.2	6.6 ± 0.5
Albumin (g/dL)				
M	3.1 ± 0.2	3.2 ± 0.1	3.0 ± 0.1	3.2 ± 0.1
F	3.6 ± 0.1	3.8 ± 0.2	3.5 ± 0.1	3.8 ± 0.4
Globulin (g/dL)				
M	3.0 ± 0.2	2.9 ± 0.2	3.1 ± 0.3	2.9 ± 0.3
F	2.7 ± 0.1	2.8 ± 0.2	2.9 ± 0.2	2.8 ± 0.3
T-Bili (mg/dL)				
M	0.11 ± 0.10	0.15 ± 0.14	0.25 ± 0.24	0.05 ± 0.05
F	0.16 ± 0.10	0.17 ± 0.20	0.17 ± 0.18	0.12 ± 0.06
Glucose (mg/dL)				
M	127.7 ± 14.8	118.4 ± 14.1	112.2 ± 14.1	105.2 ± 17.8
F	100.2 ± 12.8	101.3 ± 13.7	92.2 ± 17.4	105.2 ± 14.9
BUN (mg/dL)				
M	15.0 ± 1.6	13.4 ± 1.8	14.0 ± 1.3	14.1 ± 2.2
F	16.7 ± 3.7	15.4 ± 2.6	15.9 ± 1.3	18.3 ± 4.4
Crea (mg/dL)				
M	0.5 ± 0.1	0.5 ± 0.0	0.6 ± 0.1	0.6 ± 0.1
F	0.6 ± 0.0	0.5 ± 0.1	0.6 ± 0.1	0.6 ± 0.1
Na (mEq/dL)				
M	143.5 ± 0.8	144.1 ± 0.9	143.0 ± 0.9	145.4 ± 1.5^*∗∗*^
F	144.3 ± 1.1	143.7 ± 0.8	143.7 ± 0.9	144.9 ± 1.4
K (mEq/dL)				
M	3.6 ± 0.3	3.5 ± 0.2	3.7 ± 0.4	3.6 ± 0.3
F	3.8 ± 0.3	3.8 ± 0.3	3.8 ± 0.2	4.0 ± 0.3
Cl (mEq/dL)				
*M*	102.4 ± 1.5	102.3 ± 0.7	103.1 ± 1.2	101.5 ± 2.2
F	102.7 ± 1.4	103.5 ± 1.4	103.8 ± 1.1	104.3 ± 0.9^*∗*^
Ca (mg/dL)				
M	10.9 ± 0.2	10.8 ± 0.1	10.9 ± 0.2	11.0 ± 0.4
F	10.1 ± 0.3	10.0 ± 0.2	9.9 ± 0.2	10.0 ± 0.2
P (mg/dL)				
M	4.8 ± 0.7	5.0 ± 1.1	4.5 ± 0.5	4.8 ± 1.6
F	6.5 ± 0.8	6.2 ± 0.5	6.5 ± 0.7	6.0 ± 0.6

All values are means ± SD (*n* = 10). ^*∗*^*P* < 0.05, ^*∗∗*^*P* < 0.01 compared with control group. M: male; F: female. T-Bili: total bilirubin; and Crea: creatinine.

**Table 4:** Absolute organ weight of SD male and female rats fed with 800, 1600, and 2400 mg/kg DTTE for 90 days.

Item	Control	800 mg	1600 mg	2400 mg
Brain (g) (g/100 g b.w.)				
M	2.19 ± 0.11	2.21 ± 0.09	2.14 ± 0.05	2.16 ± 0.08
	(0.42 ± 0.04)	(0.42 ± 0.03)	(0.40 ± 0.03)	(0.42 ± 0.04)
F	2.03 ± 0.02	1.99 ± 0.02	2.07 ± 0.01	2.01 ± 0.02
	(0.73 ± 0.04)	(0.71 ± 0.06)	(0.74 ± 0.05)	(0.72 ± 0.04)
Pituitary (mg) (mg/100 g b.w.)				
M	13.3 ± 6.8	10.3 ± 1.5	10.6 ± 1.5	12.4 ± 5.1
	(2.5 ± 1.4)	(2.0 ± 0.4)	(2.0 ± 0.4)	(2.4 ± 0.9)
F	15.8 ± 2.3	17.4 ± 6.6	14.6 ± 2.8	13.2 ± 3.0
	(5.5 ± 0.8)	(6.2 ± 2.4)	(5.3 ± 1.3)	(4.7 ± 1.1)
Heart (g) (g/100 g b.w.)				
M	1.52 ± 0.13	1.52 ± 0.13	1.49 ± 0.11	1.52 ± 0.13
	(0.29 ± 0.03)	(0.29 ± 0.03)	(0.28 ± 0.02)	(0.29 ± 0.03)
F	0.93 ± 0.10	0.94 ± 0.09	0.94 ± 0.06	0.95 ± 0.10
	(0.33 ± 0.03)	(0.34 ± 0.04)	(0.34 ± 0.04)	(0.34 ± 0.03)
Liver (g) (g/100 g b.w.)				
M	12.7 ± 2.1	12.0 ± 1.6	11.8 ± 1.7	12.3 ± 1.8
	(2.4 ± 0.4)	(2.3 ± 0.3)	(2.2 ± 0.3)	(2.4 ± 0.4)
F	6.8 ± 0.5	6.7 ± 0.6	6.7 ± 0.5	7.0 ± 0.7
	(2.4 ± 0.2)	(2.4 ± 0.3)	(2.4 ± 0.3)	(2.5 ± 0.2)
Spleen (g) (g/100 g b.w.)				
M	0.82 ± 0.09	0.79 ± 0.09	0.80 ± 0.09	0.80 ± 0.06
	(0.16 ± 0.02)	(0.15 ± 0.02)	(0.15 ± 0.02)	(0.16 ± 0.01)
F	0.54 ± 0.09	0.54 ± 0.06	0.54 ± 0.07	0.50 ± 0.03
	(0.19 ± 0.03)	(0.19 ± 0.02)	(0.20 ± 0.03)	(0.18 ± 0.01)
L kidneys (g) (g/100 g b.w.)				
M	1.63 ± 0.12	1.52 ± 0.13	1.51 ± 0.11	1.55 ± 0.12
	(0.31 ± 0.03)	(0.29 ± 0.03)	(0.28 ± 0.02)	(0.32 ± 0.09)
F	0.83 ± 0.09	0.84 ± 0.08	0.85 ± 0.07	0.82 ± 0.07
	(0.30 ± 0.04)	(0.30 ± 0.03)	(0.30 ± 0.04)	(0.29 ± 0.02)
R kidneys (g) (g/100 g b.w.)				
M	1.66 ± 0.11	1.54 ± 0.12	1.54 ± 0.10	1.53 ± 0.13
	(0.31 ± 0.03)	(0.29 ± 0.02)	(0.29 ± 0.02)	(0.27 ± 0.06)
F	0.86 ± 0.09	0.87 ± 0.09	0.85 ± 0.06	0.84 ± 0.07
	(0.31 ± 0.04)	(0.31 ± 0.04)	(0.31 ± 0.04)	(0.30 ± 0.02)
L adrenal (mg) (mg/100 g b.w.)				
M	25.0 ± 1.4	29.6 ± 1.5	29.7 ± 1.7	27.3 ± 1.4
	(4.8 ± 1.0)	(5.6 ± 1.0)	(5.6 ± 0.8)	(5.3 ± 1.0)
F	31.3 ± 6.1	32.6 ± 5.5	30.0 ± 4.5	32.6 ± 6.1
	(11.2 ± 2.3)	(11.7 ± 1.9)	(10.7 ± 2.0)	(11.7 ± 2.5)
R adrenal (mg) (mg/100 g b.w.)				
M	25.9 ± 3.9	28.4 ± 4.7	27.6 ± 4.2	24.4 ± 4.0
	(4.9 ± 0.8)	(5.4 ± 0.9)	(5.2 ± 0.7)	(4.7 ± 0.8)
F	30.0 ± 6.2	31.4 ± 6.6	28.8 ± 3.8	31.1 ± 6.8
	(10.6 ± 2.0)	(11.3 ± 2.7)	(10.3 ± 1.9)	(11.1 ± 2.6)
L testis (g) (g/100 g b.w.)				
M	1.74 ± 0.13	1.72 ± 0.14	1.76 ± 0.12	1.71 ± 0.23
	(0.33 ± 0.03)	(0.33 ± 0.03)	(0.33 ± 0.04)	(0.33 ± 0.05)
R testis (g) (g/100 g b.w.)				
M	1.72 ± 0.13	1.73 ± 0.13	1.76 ± 0.15	1.70 ± 0.24
	(0.33 ± 0.03)	(0.33 ± 0.03)	(0.33 ± 0.04)	(0.33 ± 0.06)
L ovaries (mg) (mg/100 g b.w.)				
F	53.3 ± 10.1	55.6 ± 8.0	56.4 ± 7.0	47.3 ± 8.5
	(19.2 ± 4.4)	(19.9 ± 3.2)	(20.3 ± 3.9)	(16.9 ± 3.4)
R ovaries (mg) (mg/100 g b.w.)				
F	58.1 ± 7.8	57.4 ±10.2	59.5 ± 8.4	50.2 ± 7.1
	(20.8 ± 3.1)	(20.6 ± 4.0)	(21.3 ± 4.1)	(17.9 ± 2.6)

All values are means ± SD (*n* = 10). (  ): relative weight; b.w.: body weight; M: male; F: female; L: left; and R: right.

**Table 5:** Histopathological examination of SD male and female rats fed with 800, 1600, and 2400 mg/kg DTTE for 90 days.

Organ	Lesions	Group			
		Male		Female	
		H_2_O	DTTE 2.4 g/kg	H_2_O	DTTE 2.4 g/kg
Adrenal		-	-	-	-
Aorta		-	-	-	-
Brain		-	-	-	-
Brain stem		-	-	-	-
Bone		-	-	-	-
Bone marrow		-	-	-	-
Esophagus		-	-	-	-
Eye		-	-	-	-
Heart					
	Infiltration, mononuclear cell, focal, minimal to slight^2^	2/10^1^	1/10	-	-
Intestine		-	-	-	-
Kidney					
	Hydronephrosis, pelvis, focal, slight to moderate	2/10	-	-	-
	Infiltration, mononuclear cell, focal, slight	1/10	-	-	-
	Regeneration, tubule, focal, slight	1/10	-	-	-
Liver		-	-	-	-
Lung		-	-	-	-
Lymph node		-	-	-	-
Ovary		N	N	-	-
Oviduct		N	N	-	-
Pancreas		-	-	-	-
Parathyroid gland		-	-	-	-
Prostate					
	Infiltration, mononuclear cell, focal, slight	2/10	2/10	N	N
Sciatic nerve		-	-	-	-
Seminal vesicle		-	-	-	-
Skeletal muscle		-	-	-	-
Spinal cord		-	-	-	-
Stomach		-	-	-	-
	Epidermoid cyst, focal, moderate	-	-	-	1/10
Spleen		-	-	-	-
Testes		-	-	N	N
Thymus		-	-	-	-
Thyroid gland		-	-	-	-
Trachea		-	-	-	-
Urinary bladder		-	-	-	-
Uterus		N	N	-	-
Vagina		N	N	-	-

^1^Incidence: number of affected rats/number of rats were examined. ^2^Degree of lesions was graded from one to five depending on severity: 1 = minimal (<1%); 2 = slight (1–25%); 3 = moderate (26–50%); 4 = moderate/severe (51–75%); and 5 = severe/high (76–100%).

**Table 6:** Bactericidal effect of DTTE in *Salmonella* TA98, TA100, TA102, TA1535, and TA1537 tester strains.

Tester strains	Bacterial concentration	Conc. (mg/plate)/number of revertants (colony, CFU/plate)			
		0	1.25	2.5	5
TA98	10^−6^/ml	192.0 ± 11.0	195.0 ± 10.8	196.7 ± 4.6	200.3 ± 9.0
	10^−7^/ml	24.0 ± 2.2	25.7 ± 2.6	23.7 ± 3.3	25.0 ± 4.5
TA100	10^−6^/ml	209.0 ± 4.5	204.0 ± 5.4	214.3 ± 2.6	208.0 ± 7.8
	10^−7^/ml	25.7 ± 4.2	26.3 ± 3.9	27.3 ± 2.1	25.3 ± 2.6
TA102	10^−6^/ml	213.0 ± 6.7	208.7 ± 9.8	224.0 ± 7.8	221.3 ± 8.2
	10^−7^/ml	24.0 ± 2.4	25.0 ± 2.8	27.7 ± 3.4	28.3 ± 4.0
TA1535	10^−6^/ml	123.3 ± 4.1	122.3 ± 5.4	122.0 ± 9.1	126.3 ± 4.9
	10^−7^/ml	12.3 ± 2.1	10.0 ± 5.4	10.7 ± 0.5	9.3 ± 3.9
TA1537	10^−6^/ml	21.0 ± 3.3	23.7 ± 1.7	22.7 ± 3.3	21.0 ± 2.2
	10^−7^/ml	6.0 ± 0.8	8.3 ± 1.7	7.7 ± 0.5	5.7 ± 1.7

Data are expressed as the mean ± SD (*n* = 3).

**Table 7:** Revertant changes of DTTE on *Salmonella typhimurium* strains mutagenicity test in the presence and absence of S9 mixture.

Compound	Conc. (mg/plate)	S9	Number of revertants (colony/plate)				
			TA98	TA100	TA102	TA1535	TA1537
Negative control	0	−	31.7 ± 3.9	183.7 ± 7.0	268.7 ± 20.4	12.3 ± 2.1	11.0 ± 4.3
DTTE	0.3125	−	26.0 ± 1.6	186.3 ± 6.1	268.0 ± 15.6	9.3 ± 1.2	12.3 ± 2.5
	0.625	−	26.0 ± 1.6	185.3 ± 6.9	274.3 ± 21.7	10.0 ± 2.9	12.7 ± 0.5
	1.25	−	24.0 ± 2.2	180.7 ± 2.9	275.3 ± 10.3	11.0 ± 1.4	10.0 ± 2.9
	2.5	−	27.3 ± 2.1	181.3 ± 11.0	269.3 ± 12.8	10.0 ± 0.8	10.0 ± 3.3
	5.0	−	25.3 ± 1.7	182.0 ± 4.9	279.0 ± 26.5	13.3 ± 1.9	14.3 ± 2.1
4-NQO		−	172.3 ± 32.3^*∗*^				
NaN3		−		2208.0 ± 43.6^*∗*^		552.7 ± 29.8^*∗*^	
MMC		−			2113.3 ± 357.4^*∗*^		
9-AA		−					1499.3 ± 68.7^*∗*^

Negative control		+	24.7 ± 3.1	182.7 ± 7.7	309.7 ± 22.4	11.3 ± 4.5	9.0 ± 2.2
DTTE	0.3125	+	31.0 ± 1.6	177.7 ± 9.0	310.7 ± 28.5	11.3 ± 1.9	12.7 ± 3.1
	0.625	+	32.7 ± 5.2	184.3 ± 6.1	316.3 ± 21.9	9.0 ± 2.2	7.7 ± 0.9
	1.25	+	27.0 ± 2.4	183.3 ± 6.9	307.7 ± 33.5	10.0 ± 3.3	12.3 ± 3.3
	2.5	+	27.3 ± 2.1	185.0 ± 7.8	330.3 ± 29.8	12.3 ± 3.1	8.7 ± 1.9
	5.0	+	25.3 ± 1.7	186.7 ± 2.6	307.7 ± 21.3	14.7 ± 2.4	12.0 ± 1.6
2-AA		+	2858.0 ± 264.7^*∗*^	3340.3 ± 71.8^*∗*^	1359.0 ± 125.2^*∗*^	230.0 ± 6.7^*∗*^	228.0 ± 21.1^*∗*^

Data was presented as mean ± SD (*n* = 3 replicate plates); negative control for *Salmonella* strains was added with sterile reversed osmosis water; positive reagents without S-9 mix reactions were 2.5 *μ*g/plate 4-nitroquinoline-N-oxide (4-NQO) for TA98, 5 *μ*g/plate sodium azide (NaN_3_) for TA100, TA1535, and 0.5 *μ*g/plate mitomycin C (MMC) for TA102 and 50 *μ*g/plate 9-aminoacridine (9-AA) for TA1537; positive reagent with S-9 mix reactions was 5 *μ*g/plate 2-aminoanthracene (2-AA) for all tester strains. ^*∗*^Significant difference of colonies more than twofold of negative control and treated groups at *P* < 0.05.

**Table 8:** Mammalian cell micronucleus test of DTTE in mice treated with water, DTTE, or cyclophosphamide.

Treatment	Dose (mg/kg)	Number of RET	% of MN in RET	RET/NCE (%)
24 hours				
Control	-	1002.0 ± 8.5	2.0 ± 0.7	5.4 ± 0.6
DTTE	800	1006.0 ± 8.7	2.4 ± 0.2	5.9 ± 0.9
	1600	1003.8 ± 7.8	2.2 ± 0.6	5.0 ± 0.5
	2400	1005.3 ± 3.1	2.8 ± 0.5	5.6 ± 0.9
Cyclophosphamide	100^a^	987.7 ± 29.7	8.4 ± 1.0^*∗∗∗*^	2.1 ± 0.3^*∗∗∗*^
48 hours				
Control		997.8 ± 8.8	2.3 ± 0.8	5.4 ± 1.1
DTTE	800	998.0 ± 8.8	1.9 ± 0.9	4.9 ± 1.0
	1600	1000.7 ± 3.9	1.7 ± 0.7	4.7 ± 1.2
	2400	995.3 ± 7.0	1.9 ± 0.6	5.2 ± 0.7
Cyclophosphamide	100^a^	1004.7 ± 14.2	12.9 ± 2.3^*∗∗∗*^	2.0 ± 0.1^*∗∗∗*^

All values are means ± SD (*n* = 6); ^*∗∗∗*^*P* < 0.001 as compared with the control group; RET: reticulocytes; NCE: normochromatic erythrocyte; and MN: micronucleus. a: cyclophosphamide via intraperitoneal injection (10 mL/kg).
